# AI-enhanced patient-specific dosimetry in I-131 planar imaging with a single oblique view

**DOI:** 10.1038/s41598-025-09212-7

**Published:** 2025-07-08

**Authors:** Mostafa Jalilifar, Mahdi Sadeghi, Alireza Emami-Ardekani, Ahmad Bitarafan-Rajabi, Kouhyar Geravand, Parham Geramifar

**Affiliations:** 1 Department of Radiologic Technology, School of Allied Medical Sciences, Ahvaz Jundishapur University of Medical Sciences, Ahvaz, Iran; 2 Medical Physics Department, School of Medicine, Iran University of Medical Sciences, Tehran, Iran; 3 Research Center for Nuclear Medicine, Tehran University of Medical Sciences, Tehran, Iran; 4 Cardiovascular Interventional Research Center, Rajaie Cardiovascular Institute, Iran University of Medical Sciences, Tehran, Iran

**Keywords:** Planar imaging, Internal dosimetry, Dose point kernel, Artificial intelligence, Outcomes research, Biophysics

## Abstract

This study aims to enhance the dosimetry accuracy in ^131^I planar imaging by utilizing a single oblique view and Monte Carlo (MC) validated dose point kernels (DPKs) alongside the integration of artificial intelligence (AI) for accurate dose prediction within planar imaging. Forty patients with thyroid cancers post-thyroidectomy surgery and 30 with neuroendocrine tumors underwent planar and SPECT/CT imaging. Using whole-body (WB) planar images with an additional oblique view, organ thicknesses were estimated. DPKs and organ-specific S-values were used to estimate the absorbed doses. Four AI algorithms- multilayer perceptron (MLP), linear regression, support vector regression model, decision tree, convolution neural network, and U-Net were used for dose estimation. Planar image counts, body thickness, patient BMI, age, S-values, and tissue attenuation coefficients were imported as input into the AI algorithm. To provide the ground truth, the CT-based segmentation generated binary masks for each organ, and the corresponding SPECT images were used for GATE MC dosimetry. The MLP-predicted dose values across all organs represented superior performance with the lowest mean absolute error in the liver but higher in the spleen and salivary glands. Notably, MLP-based dose estimations closely matched ground truth data with < 15% differences in most tissues. The MLP-estimated dose values present a robust patient-specific dosimetry approach capable of swiftly predicting absorbed doses in different organs using WB planar images and a single oblique view. This approach facilitates the implementation of 2D planar imaging as a pre-therapeutic technique for a more accurate assessment of the administrated activity.

## Introduction

Iodine-131 is broadly applied for the treatment of benign and malign thyroid diseases as well as for neuroendocrine tumors^1–4^. Even though these indicators differ basically from one another, there are similar considerations and obstacles in patient imaging and dosimetry for both ^131^I-NaI and ^131^I- meta-iodobenzylguanidine (mIBG). Planar scintigraphy has been typically performed to evaluate the uptake and distribution of radioiodine in different organs, especially cancerous cells. It helps in optimizing the treatment to ensure the effective destruction of cancerous cells while minimizing radiation exposure to healthy tissues.

The initial proposal for the whole-body planar scintigraphy was made by Thomas et al., using the calculation of the geometric mean (GM) from anterior and posterior opposing views with effective attenuation correction (AC)^5^. However, a primary shortcoming of this approach was the potential for overlap in organ-organ or organ-background in the projections, that could result in inaccuracies in estimating the activity for the organs of interest^6^. Not to mention that performing accurate organ segmentation is another important challenge in planar quantification.

Employing Single Photon Emission Computed Tomography (SPECT) offers 3D spatial data and addresses the issue of organ overlap which can be a critical stage in performing 3D voxel-level dosimetry^7^. Furthermore, contemporary dual-modality SPECT/CT scanners present an enhanced attenuation map from CT to facilitate AC for SPECT reconstruction leading to the improvement of organ segmentation in radionuclide therapy^8^. However, planar scintigraphy, on the other hand, is a more traditional imaging technique and is likely to be available in a large number of nuclear medicine centers. Not to mention that, using planar imaging, contouring lesions and organs can be simply performed with common software tools available in a given nuclear medicine system, unlike SPECT/CT based dosimetry that requires specialized software.

Internal dosimetry has gained increased significance in recent years due to the rising focus on personalized healthcare and targeted radiopharmaceutical therapy^9^. In clinical trials, monitoring patient doses has typically relied on simplified models, such as the ones that are derived from the Medical Internal Radiation Dose Committee (MIRD) methodology^10^. The conventional MIRD method has been carried out based on organ dosimetry offering dose distributions for the general population utilizing time-integrated activity and radionuclide S-values, showing the average radiation dose received to a target tissue for each radioactive decay in a source tissue. The main demerit of this approach is associated with the presumption of uniform activity distribution in organs and that it cannot consider patient-specific geometries. To address the variations of anatomical characteristics among patients, the organ-based dosimetry model was subsequently developed by patient-specific computational methods^11–13^. In this regard, another important dosimetry method is dose point kernel (DPK)^13,14^ representing a radial absorbed dose in a homogeneous water medium when an isotropic point source is located at the center^15–17^. Nevertheless, the DPK method can be used for lesions in homogeneous tissue media since the medium heterogeneity is not taken into account in these techniques. To address the abovementioned limitations of dosimetry methods, direct Monte Carlo (MC) as a voxel-based dosimetry approach has been introduced, taking into consideration the heterogeneous medium and non-uniform activity distribution. Up to the present, the direct MC approach can provide an accurate voxel-based dosimetry depending on the accuracy of the models and it is considered as a reliable dose estimation technique in clinical practice^16,18^. Nevertheless, the direct MC method demands substantial computational resources and time. That is why it is seldom employed in routine clinical trials. Consequently, there is a need to provide a rapid voxelized dosimetry method considering heterogeneous activities and medium distributions^19^.

Deep learning has recently surfaced as a potential model in the field of image processing, demonstrating superb presentation in comparison with routine techniques in the analysis of medical images in positron emission tomography (PET) and SPECT imaging, containing attenuation and scatter corrections^20,21^, and automated image segmentation^22,23^. Deep learning techniques have been recently utilized for estimating radiation dose. Mardani et al.^24^ employed a multi-layer convolutional neural network structure to estimate dose distribution in external beam radiation therapy. Nguyen et al.^25^ applied a U-Net deep learning approach to optimize clinical treatment planning while decreasing the computational time. In another study, Ma et al.^26^ utilized a conventional neural network to supply isodose characteristics for treatment planning of modulated arc therapy. Kearney et al.^27^ implemented deep learning for dose estimation in prostate stereotactic body radiotherapy patients. It is important to mention that for efficient training of a deep learning technique, providing an explicit easy-to-understand ground truth is a crucial aspect^28^. In the aforementioned studies, the ground truth data were acquired from MC dosimetry for the network training that might contain uncertainty due to the simplification involved in physical models^29^. Lee et al.^30^ could overcome this issue by employing a U-Net deep neural network, obtaining the ground truth from direct MC simulation. They input CT and static PET images into the network to estimate a 3D dose distribution map. In another study, Gotz et al. used a modified U-net network for dose prediction of individuals who were administered ^177^Lu-PSMA in which the training datasets included CT images, MIRD-based voxelized dose distribution map acquired from SPECT images, and direct MC data as the ground truth. It was reported that when employing a four-core and 16 GB RAM CPU, the duration for an MC simulation of a whole body surpasses 4700 h^31^. Nevertheless, there have been recent calls for GPU-based MC simulations to address this issue^32,33^.

This study aims to improve the dosimetry accuracy in ^131^I whole-body (WB) planar imaging by exploring different DPKs as well as incorporating an additional oblique view, and comparing results with MC-based SPECT/CT dosimetry as the ground truth. The study employs artificial intelligence (AI) to estimate dose distribution in planar imaging utilizing a training dataset comprising patients’ demographic data (Body Mass Index (BMI) and age), anterior and posterior image counts, body thickness from the oblique view, S-value related to each tissue obtained from DPK, the effective attenuation coefficient for tissues, and the corresponding dose values as the outputs. This approach offers dual benefits: firstly, it incorporates modified planar imaging, a more prevalent method than SPECT/CT, and secondly, it significantly reduces processing time compared to MC simulations. While previous efforts have focused on enhancing the dosimetry of planar imaging, this research utilizes AI algorithms on planar imaging data and one single oblique view for estimating organ doses. Some recent studies have aimed to improve radioiodine therapy dosimetry^34,35^. For instance, Georgiou et al.^34^ describes the difficulty of the conventional MIRD-based dosimetry protocol involved imaging and blood sampling at 4, 24, 48, 72, and 96 h post-administration of a tracer activity of ^131^I and explores the use of AI in simplifying and optimizing radioiodine therapy dosimetry through predicting the maximum permissible activity^34^. However, given the importance of determining the exact dose values received by different organs, the present study considered imaging data to improve the prediction of the pixel-based absorbed doses by each tissue.

## Materials and methods

### Patients

Seventy patients including 40 suffering from thyroid cancers and 30 with neuroendocrine tumors underwent planar and SPECT/CT imaging modalities following an intravenous administration of ^131^I-NaI and ^131^I-MIBG respectively. Patients with thyroid cancer first underwent thyroidectomy, and to remove remnant cancerous cells, radioiodine therapy was performed. To provide the patient body thickness and the thickness of each organ, an additional 45-degree oblique view was acquired during the planar scan. Clinical data including age, sex, disease staging, and treatment received were obtained from the patient database of our center. The study protocol was approved by the Ethics Committee of Iran University of Medical Sciences (IUMS) (IR.IUMS.FMD.REC.1401.059) and followed the ethical principles of the Declaration of Helsinki. An informed consent was also obtained from all patients and their legal guardians before planar and SPECT/CT scans.

### Imaging protocols

Planar and SPECT/CT images were obtained using a Symbia T-Series Siemens Healthlineers, Germany. Planar ^131^I whole-body imaging was performed 24h, 72h, and 168h after the administration of ^131^I radionuclide capsule (between 3700 and 7400 MBq (100 and 200mci)) in anterior, posterior, and oblique projections using the dual-head scanner with a matrix size of 1024 × 256.

The SPECT datasets were performed 72h after radioiodine administration and obtained with 64 projections of 20 s per detector over 360°, using a high-energy collimator. The ordered-subsets expectation maximization (OSEM) technique was performed (four iterations/eight subsets) for image reconstruction. The reconstructed SPECT images included dimensions of 64 × 64 × 64 with a voxel size of 9.59 × 9.59 × 9.59 mm^3^. The CT images were acquired with the dimension of 512 × 512 × 79 mm^3^ with a voxel size of 0.976 × 0.976 × 5 mm^3^. The CT images were then down-sampled to fuse with the SPECT data. The focused regions were manually drawn on the SPECT/CT and planar images for heart, kidneys, liver, the upper part of lungs, the middle part of lungs, the lower part of lungs, spleen, thyroid, salivary glands, and tumor. To more accurately apply attenuation correction (AC) in the lung, it was divided into 3 parts including upper, middle, and lower parts. The upper part of the lung is surrounded by thick rib bone material, and the thickness was decreased in the lower parts. Therefore, according to the values of HU in CT images, the effective AC was separately performed for each part of the lung. The regions in focus were segmented by a nuclear medicine physician and affirmed by another experienced nuclear medicine physician.

### Planar quantification

We used the conventional planar method according to the evaluation of the geometric mean (GM) of two opposing views and the effective AC^5^. The activity (A) in the ROI was calculated using Eq. (1):

1 $$\text{A}=\sqrt{\frac{{{\text{I}}_{\text{A}}\text{I}}_{\text{B}}}{{\text{e}}^{-{\upmu }_{\text{e}}\text{T}}}}\times \frac{1}{\text{C}}$$

where I_A_ and I_B_ respectively represent the counts in the ROI on the anterior and posterior images, µ_e_ denotes the effective attenuation coefficient and can be considered as the average of all attenuation coefficients in each region, T stands for the body thickness obtained from an oblique view of the planar scan, and C is the calibration factor. To optimize the planar quantification, we used CT-based Hounsfield unit data to determine µ_e_ for each organ. To determine each tissue attenuation coefficient, the direction through which the gamma ray passes in the body should be considered. The calibration factor which is needed for both SPECT and planar quantification was determined using two methods according to MIRD 16^36^ and MIRD 24^37^; (1) a planar scan of a known activity placed in a Petri dish in the air wherein 40.7 MBq (1.1 mci) of ^131^I was uniformly drowned into 40 ml water at 10 cm from the camera. (2) another method consisted of planar and SPECT scans of a point-like source (syringe needle) with 46.62 MBq (1.26 mci) in the air at 10 cm from the camera.

### Partial volume effect (PVE)

To more accurately provide activity quantification, partial volume effect (PVE) was evaluated for planar and SPECT/CT imaging modalities using Carlson phantom that contained 7 spheres of diameters 7.3, 9.2, 11.4, 14.3, 17.9, 22.4, and 29.9 mm filled with a 1276.5 kBq/ml (0.0345 mci/ml) ^131^I solution with the background activity concentration of 161.16 kBq/ml (4.3 × 10^–3^ mci/ml).

### Accumulated activity

Evaluation of the accumulated activity was performed through calculating the area under the time-activity curve fitted from three-time point planar scans including 24h, 72h, and 168h after the radioiodine administration. A biexponential decay function was fitted to the counting rate kinetics of different tissues and tumors using ordinary least-squares regression^38^. This choice is supported by both practical and scientific considerations. Analysis of prior studies, including Jackson et al. (2020), shows that three time points can suffice for accurate time-integrated activity estimation when leveraging population-based pharmacokinetic models or mixed-model fitting, particularly for radiopharmaceuticals like ^131^I^39^. By using three time points, we balanced accuracy with patient safety and imaging feasibility, as further validated in a recent publication^38^.

### Dose point kernel (DPK)

We applied the pixel-based kernel convolution approach to evaluate S-value (Gy/Bq.s) using the Geant4 Application for Emission Tomography (GATE) v.8.2 simulation setup. DPKs were determined for each organ separately while the ^131^I point source was placed in the middle of the heart, kidneys, liver, lung, spleen, thyroid, salivary glands, and tumor.

**Fig. 1:**
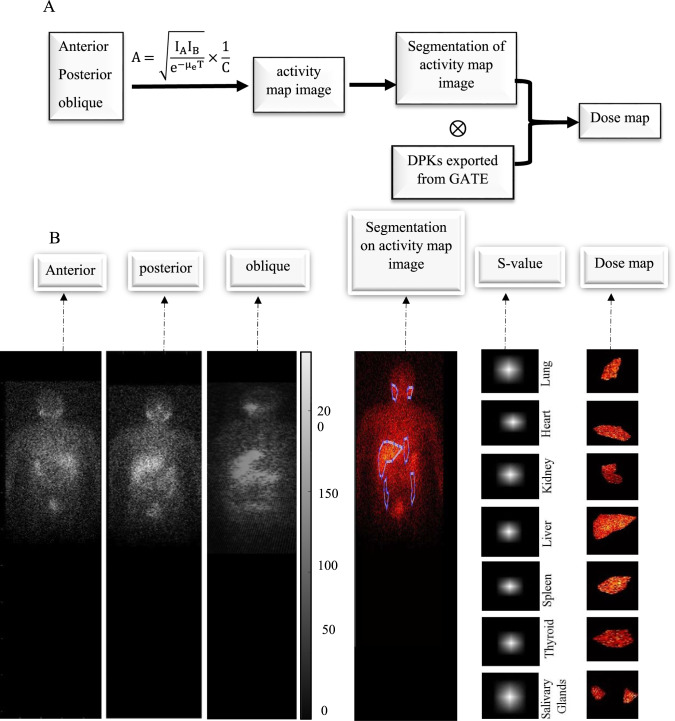
(**A**) The flowchart of patient dosimetry using the DPK method. (**B**) The process of the multiple DPKs method. The code in MATLAB first showed anterior, posterior, and 180 flip posterior planar images. Thereafter, these two images were overlapped using the GM method and converted to the activity map. In the next step, the DPK was convoluted into the activity of each organ to provide the dose map.

Figure 1 illustrates the process of the multiple DPKs method with nine different DPKs. First, DPKs with various densities were pre-calculated with the GATE software. The ^131^I point source was located at the center pixel and 10^9^ primary particles were generated. The pixel size was 2.3976 × 2.3976 mm^2^, which was similar to the planar image pixel size. The kernel size was set to 9 × 9. Care was exercised for the size of the kernel to adequately cover the dose range to prevent underestimation of the absorbed dose. The organs of interest were segmented in the planar images and image counts were determined and converted into the activity using the calibration factor. Thereafter, the cumulated activity (Ã) (MBq·s) was convoluted with the kernel to produce the output dose (D)(Gy) as indicated by Eq. (2). The basic assumption is that the majority of organ energy depositions originate from self-absorption. We developed a code in MATLAB which could run anterior, posterior, oblique, and also GM images. The MATLAB code facilitated planar image preprocessing to dosimetry, integrating activity quantification, semi-automatic segmentation, thickness computation, and dose mapping. It imported anterior and posterior planar images (.mhd format), applied GM correction after rotating the posterior image 180 degrees, and converted counts to activity using calibration coefficients, following MIRD Pamphlet 24^37^. Organ thickness was measured interactively from the 45-degree oblique view using cursor-based tools^40^. Semi-automatic segmentation, based on region-growing algorithms^41–43^ allowed user-guided organ delineation. The segmented region was convolved with a point-source kernel exported from GATE to generate organ-specific dose maps, as described by^8,44,45^. This workflow ensured accurate activity and dose estimation in clinical implementation.

2 $${\text{D}} = {\tilde{\text{A}}} \otimes {\text{DPK}}$$

### Direct MC approach: GATE MC simulation setup

GATE version 8.2 Monte Carlo simulation toolkit was employed for the simulation of direct MC and DPK pre-calculation^46,47^. Several previous studies validated the dosimetric accuracy of the GATE toolkit^47–49^.

The obtained CT and SPECT images were adjusted to show the same matrix and voxel size and they were utilized as the input data. CT images were segmented to generate a binary mask image for each region and imported into the GATE code to simulate the voxelized phantom, with Hounsfield units being transformed into a 3D density map containing corresponding elemental compositions using HU-to-density calibration curve according to the formerly published database^50^. SPECT images were utilized for the simulation of the voxelized ^131^I source distributions and the dose prediction was performed using the “DoseActor” in GATE^47^. In case of MC simulation, voxelized source simulation with 10^9^ particles was used to provide less than 0.05 uncertainty with the cut off energy of 1 keV for beta and 5 keV for gamma rays. In this study, the direct MC approach using the GATE toolkit was considered the ground truth.

### Artificial intelligence

In this study, we implemented machine learning and deep learning models in MATLAB (vesion 2022b) to enhance nuclear medicine dosimetry, using the Statistics and Machine Learning Toolbox (v 13.2) and Deep Learning Toolbox (v 14.2). The models include: Support Vector Regression model (SVR) with radial basis kernel for activity prediction^51^, Decision Tree^52^, Linear Regression for dose estimation^53^, Multilayer Perceptron (MLP) for non-linear regression^54^, Convolutional Neural Network (CNN)^55^, U-Net^31^ for image-based dose mapping. The codebase preprocesses planar images, performs semi-automatic segmentation, computes tissue thickness, and generates dose maps via convolution. For training the model, anterior and posterior planar image counts, body thickness estimated from the oblique view image, patient BMI and age, S-value related to each tissue obtained from DPK, and the effective attenuation coefficient of each tissue were imported as input to the deep machine learning methods. The multilayer perceptron (MLP) network was used with the Bayesian regularization algorithm architecture composed of 3 different hidden layers comprising 6 neurons in each layer. The SVR approach was performed with a comprehensive hyperparameter tuning process using Bayesian optimization. This procedure explored the following ranges: kernel functions tested: ‘linear’,’ rbf’, ‘polynominal’, and ‘sigmoid’, box constraint (C) values of {0.01, 0.1, 1, 10, 100}, kernel scale (gamma) values (for non-linear kernels) of {0.01, 0.1, 1, 10}. Bayesian optimization was employed to efficiently explore the parameter space and minimize the prediction error. As a result of this optimization, the best -performing SVR model used the following configuration: kernel: ‘rbf’, C = 100, gamma = 10 which provided the lowest error. Decision tree approach was performed with a hyperparameter tuning process using Bayesian optimization. This process was performed with: min leaf size of [1,50], max num split of [2, 200], split criterion of {MAE, MSE, and RMSE}, and predictor selection of {‘allsplits’, ‘curveture’}. According to the results, the minimum error was obtained for min leaf size of 5, max num splits of 20, predictor selection of curvature, and surrogate: ‘on’. The linear regression in this study used the least square method with a limited-memory Broyden–Fletcher–Goldfarb–Shanno (BFGS) optimization algorithm and maximum iteration of 1000 to approximate the solution. The Convolution neural network (CNN) was used with 3–5 layers, kernel size of (3, 3), tanh, softplus, ReLU and linear activation function, learning ratee of 0.1, 0.01, 0.001, and dropout rate was set between 0.2 and 0.5. Moreover, batch normalization was applied after each convolution layer to normalize the inputs. The best result was achieved with a CNN of 3 layers and Relu activation function. For U-Net algorithm, the activation function of ReLU and linear, Adam optimizer, learning rate of 0.001 and 0.001, batch normalization of 32 were used and the dropout rate was set between 0.2 and 0.3.

### Data processing

The implemented pipeline in MATLAB for regression-based dose prediction comprised six main steps. First, data acquisition involved importing structured numerical features such as demographics, image counts, and attenuation coefficients from .mat and .csv files. Next, data cleaning addressed missing values, with target doses excluded and non-target features imputed using linear interpolation or mean imputation, while outliers were removed based on z-score filtering. Input features were then normalized, with z-score standardization for most and min–max normalization for imaging-derived features. The dataset was split into 75% training and 25% testing sets using a fixed random seed for reproducibility. All preprocessing steps were encapsulated in MATLAB functions, and parameters (such as normalization constants) were saved for consistent application in future evaluations. Finally, the regression model was trained with loss functions like MSE and MAE, and performance was validated using these metrics. The process was carried out in MATLAB (version 2022b) and version-controlled with Git to ensure reproducibility.

### Quantitative and statistical analysis

Test and train data regression, mean absolute error (MAE), mean squared absolute error (MSE %), and root MSE (RMSE) were determined between dose maps of the reference and prediction where ${\text{y}}_{\text{i}}$ showed the dose value calculated by the AI algorithm and $\widehat{\text{y}}$ represented the reference dose (Eq. 3–5). All these three metrics are computed across the image volume and reported as global evaluation scores^56–58^. For each organ, test MSE and MAE were also separately calculated. Having selected the model that more accurately suited the data, for external validation, after checking the normality of the data using Shapiro–Wilk test, a paired t-test and a Wilcoxon test were respectively employed to compare the parametric and nonparametric absorbed organ doses of the ground truth and the optimized AI approach.

3 $${\text{ MAE}} = \frac{1}{{\text{N}}}\mathop \sum \limits_{{{\text{i}} = 1}}^{{\text{N}}} \left| {{\text{y}}_{{\text{i}}} - {\hat{\text{y}}}} \right|$$

4 $${\text{ MSE}} = \frac{1}{{\text{N}}}\mathop \sum \limits_{{{\text{i}} = 1}}^{{\text{N}}} \left( {{\text{y}}_{{\text{i}}} - {\hat{\text{y}}}} \right)^{2}$$

5 $${\text{ RMSE}} = \sqrt {\frac{1}{{\text{N}}}\mathop \sum \limits_{{{\text{i}} = 1}}^{{\text{N}}} \left( {{\text{y}}_{{\text{i}}} - {\hat{\text{y}}}} \right)^{2} }$$

## Results

### PVE results

The planar and SPECT/CT images of the point source and Petri dish (non-point source) were acquired three times during the calibration process. The results were also compared with the initial calibrated values mentioned in the brochure of the instruments. According to the results, the planar sensitivity ranged between 90 and 92 cpm/μCi (2.43 and 2.48 cpm/kBq) while the amount of SPECT sensitivity fell between 123 and 125 cpm/μCi (3.32 and 3.37 cpm/kBq). According to the results, the RC value for SPECT/CT ranged between 0.83 and 1.065; however, using planar imaging led to the identification of RC from 0.78 to 1.09. More detailed information about the PVE and device calibration are available in our recent publication^59^.

### Dosimetry results

#### DPK and MC

**Fig. 2:**
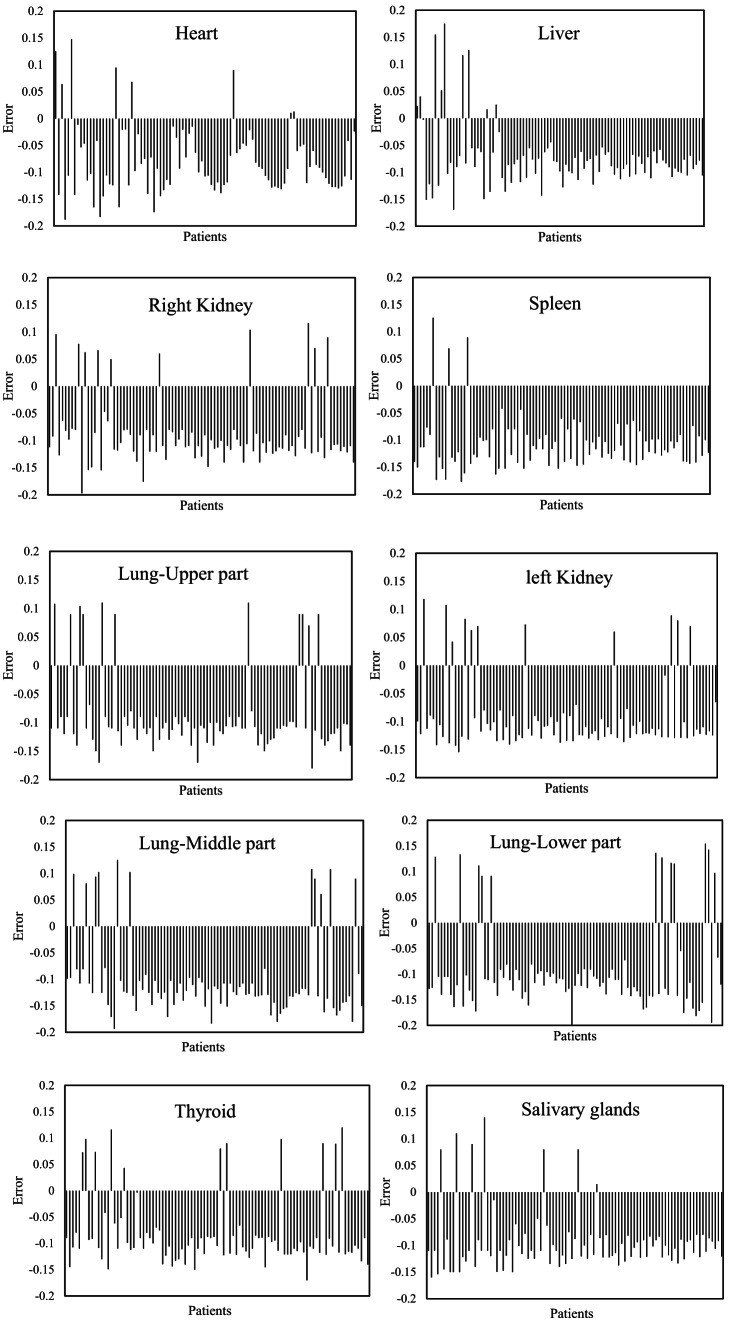
The figure illustrated the amount of dose estimation error using DPK as compared with the MC method in different organs ($\frac{DPK-MC}{MC} \times 100$).

Figure 2 presented the amount of dose estimation error using DPK as compared with the MC method in different organs. As shown in the figure, the lowest dose estimation errors were observed in the heart and liver with 7.9% and 7.3% respectively; however, the largest dose errors were identified in the spleen and middle part of the lung with 11% and 10.29%. Moreover, the largest variations in dose estimation values were observed in different parts of the lung and thyroid.

### Comparing AI and MC results

**Fig. 3:**
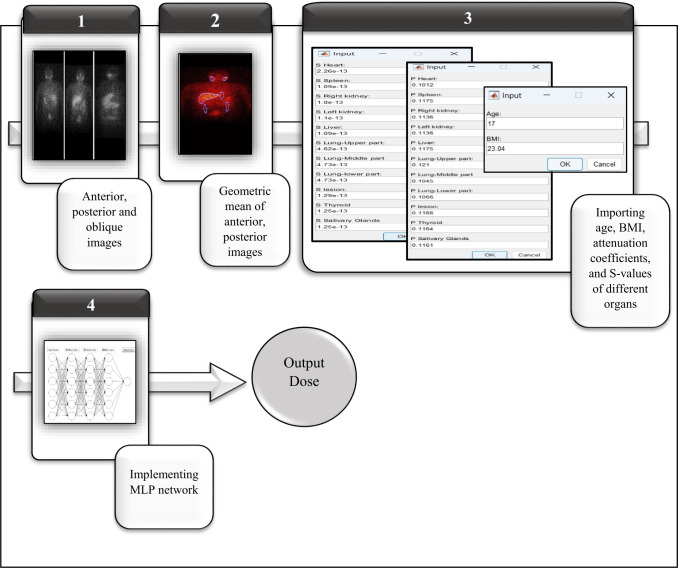
The process of applying the AI approach (for example, MLP network) on the data of DPK dosimetry of 2D planar images of the patients.

Figure 3 shows the process of applying the AI approach to the data of DPK dosimetry of 2D planar images of the patients. In the figure, the example of using an MLP network with three hidden layers (each layer contains 6 neurons) is represented. Table 1 exhibits the results of using four different machine and deep learning algorithms including MLP, decision tree, linear regression, SVR, CNN, and U-Net. The MLP network was tested for different numbers of hidden layers and neurons. In this regard, the optimal MLP model was achieved using [6 6 6] layers with the train MSE of 0.61 and MAE of 0.34. Moreover, the test MSE and MAE of the network were 2.69 and 1.56 respectively. In this regard, the validation strategy in internal validation included the hold out method, where 25% of the data was used as validation during the training. Moreover, hyperparameter tuning was performed using Adam optimizer tested with the learning rates of 0.0001,0.001, and 0.01, and also batch normalization in each layer with the batch size of 50 to avoid out of memory error with the back propagation strategy and the activation function of RELU and Leaky RELU. According to the results, the lowest MSE, MAE, and RMSE values were found for the learning rate of 0.001, and activation function of Leaky RELU wherein the α = 0.01.

**Table 1:** Results of MAE, MSE, and RMSE for different AI algorithms including MLP, decision tree, linear regression, SVR, CNN, and U-Net.

Tissue	AI	MSE	MAE	RMSE
Heart	MLP	4.02	2.00	2.00
	Decision tree	5.16	2.18	2.27
	Linear regression	6.08	2.41	2.46
	SVR	6.12	2.43	2.47
	CNN	8.42	2.92	2.76
	U-Net	8.76	3.27	3.11
Liver	MLP	2.83	1.62	1.68
	Decision tree	3.66	1.78	1.91
	Linear regression	4.38	1.95	2.09
	SVR	4.30	1.90	2.07
	CNN	6.78	2.65	2.79
	U-Net	7.12	2.88	2.92
Spleen	MLP	8.42	3.23	2.90
	Decision tree	8.66	3.39	2.94
	Linear regression	10.27	3.66	3.20
	SVR	9.88	3.57	3.14
	CNN	13.37	4.67	4.44
	U-Net	14.58	4.81	4.65
Left kidney	MLP	4.82	2.18	2.19
	Decision tree	4.85	2.2	2.20
	Linear regression	5.72	2.32	2.39
	SVR	6.43	2.47	2.53
	CNN	8.64	3.28	3.15
	U-Net	8.77	3.41	3.26
Right kidney	MLP	5.76	2.65	2.40
	Decision tree	5.80	2.72	2.40
	Linear regression	6.87	2.86	2.62
	SVR	6.66	2.80	2.58
	CNN	8.92	3.53	3.41
	U-Net	9.24	3.86	3.68
Lungs- upper part	MLP	5.95	2.63	2.43
	Decision tree	6.33	2.77	2.51
	Linear regression	6.40	2.81	2.52
	SVR	6.90	2.92	2.62
	CNN	9.67	3.67	3.51
	U-Net	10.28	3.88	3.72
Lungs- middle part	MLP	6.37	2.78	2.52
	Decision tree	6.85	2.87	2.61
	Linear regression	7.60	2.96	2.75
	SVR	7.50	2.94	2.73
	CNN	10.48	3.86	3.69
	U-Net	11.17	3.98	3.83
Lungs- lower part	MLP	4.12	2.18	2.02
	Decision tree	4.20	2.37	2.04
	Linear regression	4.35	2.39	2.08
	SVR	4.33	2.31	2.08
	CNN	6.72	2.83	2.46
	U-Net	7.37	2.98	2.74
Thyroid	MLP	3.90	1.96	1.97
	Decision tree	3.90	2.09	1.97
	Linear regression	4.80	2.16	2.19
	SVR	4.62	2.13	2.14
	CNN	8.22	3.18	3.02
	U-Net	8.61	3.35	3.18
Salivary Glands	MLP	8.26	3.22	2.87
	Decision tree	9.70	3.47	3.11
	Linear regression	8.70	3.26	2.94
	SVR	8.40	3.30	2.89
	CNN	12.36	4.28	4.07
	U-Net	12.54	4.43	4.22

AI: Artificial Intelligence. MSE: Mean Square error. MAE: Mean Absolute error. RMSE: Root Mean Square error. MLP: Multi-layer perceptron. SVR: Support Vector Machine.

The Bayesian decision tree method was used reaching Maximum objective evaluations of 30. The training MSE and MAE were respectively 1.2 and 0.89 while the test MSE and MAE were 2.81 and 1.45 for the decision tree approach. The Bayesian SVR approach could determine the training MSE and MAE of 1.8 and 1.3. Moreover, the test MSE and MAE were 3.88 and 2.1 respectively. The least square method was used to predict the output dose using the linear regression algorithm. The training MSE and MAE were 1.6 and 1.16 respectively while the test MSE and MAE were 3.27 and 1.86 for the linear regression technique. For CNN algorithm, the training MSE and MAE were 4.24 and 2.03 respectively while the test MSE and MAE were 6.72 and 2.86. Moreover, the training MSE and MAE were 6.65 and 2.98 respectively while the test MSE and MAE were 9.38 and 3.21 for U-Net technique.

As displayed in the table, the dose values using MLP in all organs were predicted with lower MSE and MAE in comparison with other methods. The MLP network provided the best performance in the liver with an MSE of 2.83 whereas the largest error was observed in the spleen and salivary glands.

**Fig. 4:**
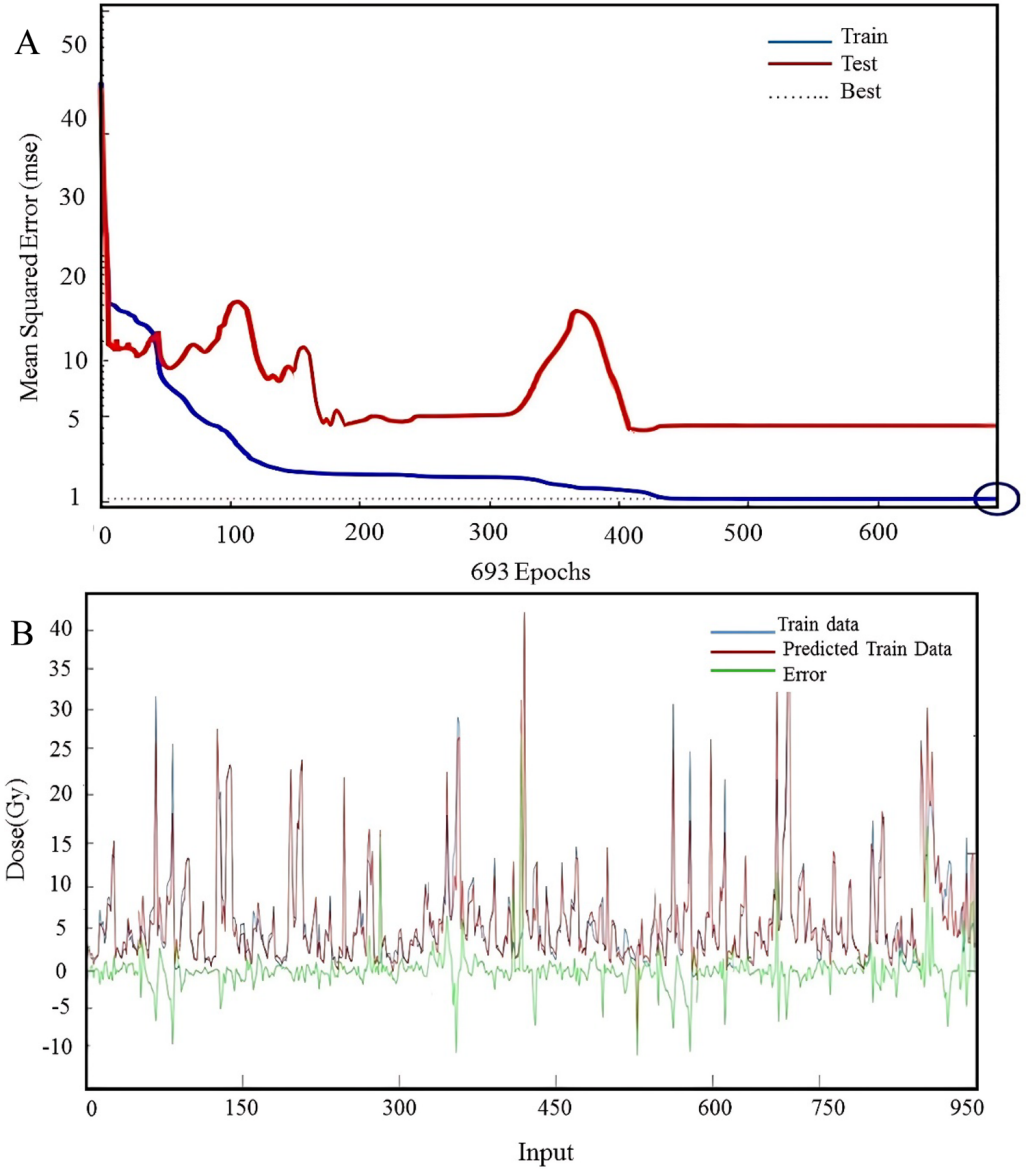
(**A**) The amount of train and test MSE for each epoch in the MLP algorithm. (**B**) The figure showed the values of train data (blue), predicted data (red), and the error (green) during the MLP model.

Since the best results were acquired using the MLP method, the output of regressions for MLP was reported. Figure 4 demonstrates the learning performance and prediction accuracy of the MLP model. Figure 4a illustrates the variations of train and test MSE values for each epoch in the MLP algorithm. As shown in the figure, the best MSE was obtained in epoch 693. The decrease in MSE over the epochs for both training and testing datasets indicates that the model is successfully learning and generalizing. The point marked ‘Best’ shows the optimal epoch with the lowest test error, reflecting the best generalization performance. Figure 4b illustrates the distribution of absolute errors between train and predicted data during the MLP model. In fact, it highlights the predicted dose values compared to actual training data, with the error plotted to show the accuracy of predictions.

**Fig. 5:**
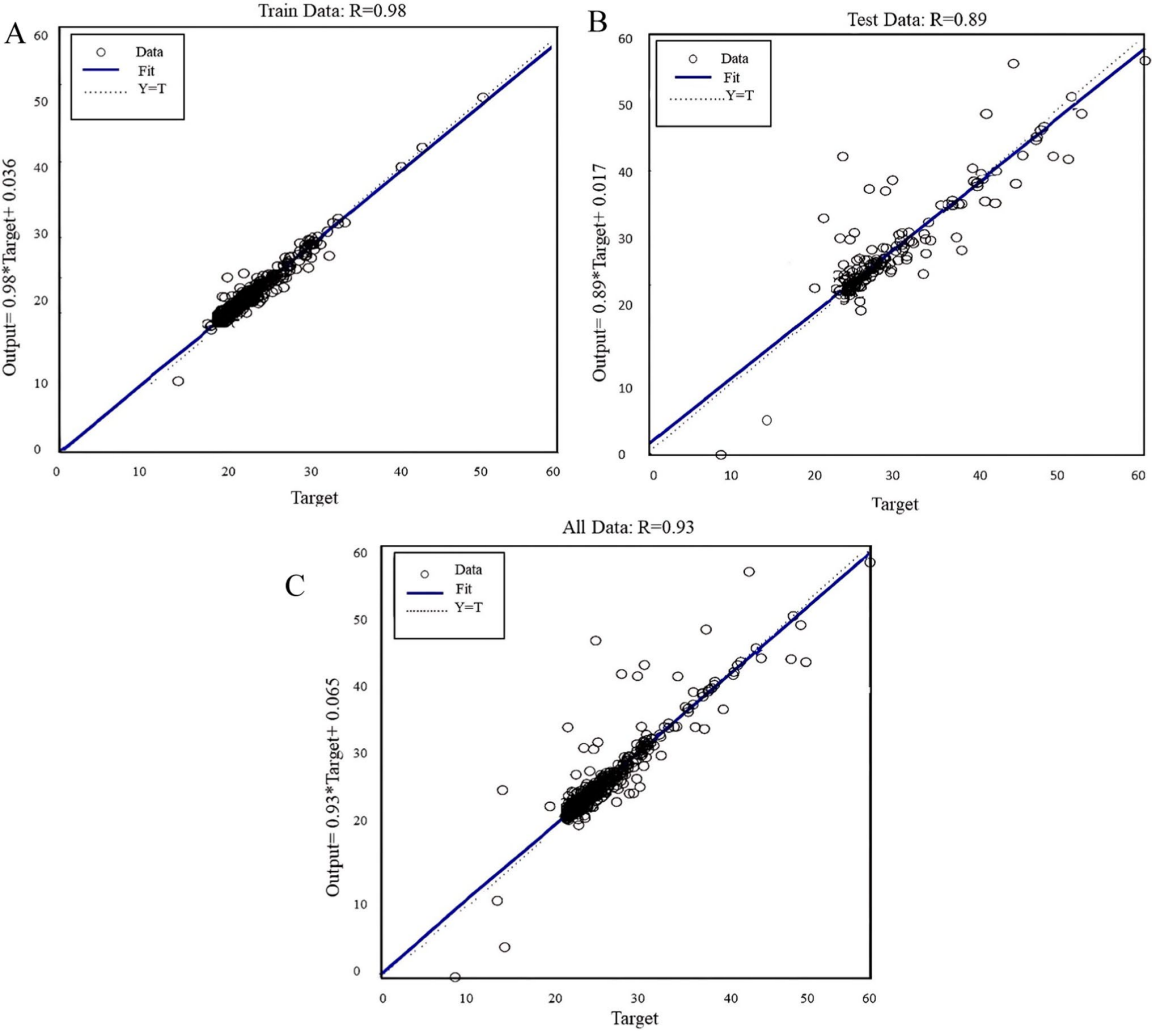
Regression of (**A**) train, (**B**) test, and (**C**) all the data of the estimated dose using the MLP algorithm.

According to the results, the best regression values are obtained using MLP algorithm. Figure 5 illustrates the regressions of train, test, and all data between output and target values using the MLP algorithm which were 0.98, 0.87, and 0.95 respectively.

### External validation

Since the MLP approach was more accurately fitted to the data, we only performed external validation for the MLP network. In this regard, for external validation, the MLP estimated doses for different organs of 23 new patients were studied and compared with MC by administering a paired t-test and a Wilcoxon test for nonparametric data. Table 2 summarizes the data of paired t-test and Wilcoxon for comparing the dose value of MC and MLP approaches.

**Table 2:** Results of paired t-test and Wilcoxon for comparing the dose value of MLP and MC techniques.

Organ	Paired t-test or Wilcoxon (MLP and MC) (*P* value < 0.05)	Percentage of error between MLP and MC
Heart	0.042	− 12.2%
Liver	0.080	7.4%
Spleen*	0.025	− 14.8%
Left Kidney	0.065	− 8.1%
Right Kidney	0.072	− 8.0%
Lung- upper part	0.032	− 13.6%
Lung- middle part	0.034	11.2%
Lung- lower part	0.036	− 12.9%
Thyroid*	0.092	6.1%
Salivary glands*	0.046	− 9.3%

*: Wilcoxon.

MLP: Multi-layer Perceptron. MC: Monte Carlo.

**Fig. 6:**
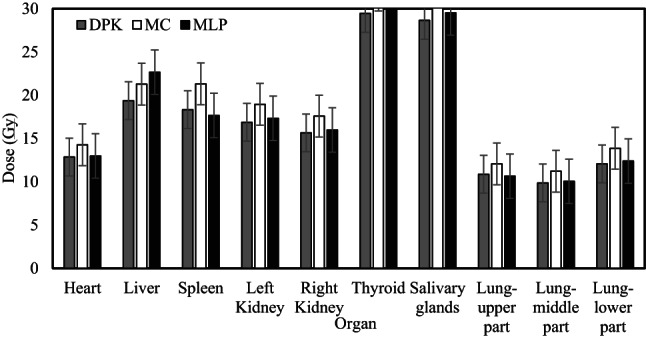
Dose values of DPK, MC, and MLP methods. DPK: Dose Point Kernel, MC: Monte Carlo, MLP: Multilayer Perceptron.

As illustrated in Fig. 6, the best dose estimation through the MLP network was performed in the thyroid; nevertheless, the worst dose estimation was achieved in the spleen. According to the results, there was no significant difference between the MC and MLP outputs for the liver, left and right kidneys, and thyroid. However, differences of -14.84%, -13.6%, and -12.9% were observed between the results of MLP and MC for the spleen, the upper part and lower part of the lungs respectively.

## Discussion

In this study, we proposed a DPK-based model to provide personalized radiation dose quantification for planar imaging. The multiple DPK approach was implemented using 9 different kernels on real patient images and compared with the direct MC approach to validate its dosimetric accuracy. As an initial step, we attempted to modify the dosimetry process of planar imaging. The main limitation for accurate dose estimation in planar imaging is associated with the identification of each tissue thickness as well as its attenuation coefficient. Estimating the attenuation coefficient was performed using the patient’s CT images. An additional oblique view of planar imaging improved the process of estimating tissue thickness. The 45-degree oblique view provides sufficient angular diversity to resolve anatomical overlap in anterior–posterior views, improving organ depth and body thickness measurements critical for accurate activity quantification. Compared to lateral views, the 45-degree angle minimizes patient repositioning and discomfort while aligning closely with CT-derived reference thicknesses, as validated in our preliminary tests. This angle thus balances accuracy and feasibility without extending scan time. Using two interactive cursors in our MATLAB-based software, we measured the apparent body thickness along the path of the oblique view. This measurement was then incorporated into the attenuation correction model according to Eq. 1, where tissue thickness (T) is a key variable. It is worth noting that prior to initiating the study, we investigated the body thickness using lateral and 45-degree oblique views, and the results were compared with the reference values derived from CT images. The results demonstrated that the 45- degree oblique view yielded measurements more closely aligned with the actual body thickness. Accordingly, the 45- degree oblique view was selected for use in the present study. Moreover, we used organ-specific DPKs to more accurately determine S-value. Another issue in applying DPK was related to the kernel size which was examined for 3 × 3, 5 × 5, 7 × 7, 9 × 9, 11 × 11, and 13 × 13 and compared with the MC. According to the results, the 9 × 9 kernel provided the closest dose values to the MC. The results could confirm the quantification error of below 13% for different organs. The lowest dose estimation errors were observed in the heart, liver, and thyroid while the largest dose errors were identified in the spleen and the middle part of the lungs. The calculated absorbed-dose values may therefore be sensitive to the imaging modality used (planar or SPECT imaging), any filtering employed, and the strategies used for image segmentation and partial-volume correction. In this regard, for each tissue, one nuclear medicine physician performed organ segmentation, and it was then confirmed or modified by another physician. Moreover, for background subtraction, the mean pixel activity in the ROI was subtracted by the mean pixel activity in the background ROI. It should be noted that the validation of the MC dosimetry results was performed through comparing the data of a Carlson phantom experiment with the simulation output. In this regard, after simulating the phantom geometry and imaging system, the simulation was repeated for 3 times using 10^9^ particles and the uncertainty was set to less than 5%. According to the validation results, the error percentages of the MC simulation were 5%, 7%, and 5% for each test and also the accuracy value of the MC simulation was found to be 94.33%. Consequently, though MC simulation is assumed as a ground truth method, the potential biases in segmentation, attenuation correction, and modelling assumptions might affect the MC results.

For improving planar quantification, Sjogreen et al. used the attenuation map obtained from integrated CT images in combination with the anterior–posterior direction to assist in defining the volume and performing background and overlapping tissue correction. The results confirmed that the inaccuracies in estimating the activity for the majority of tissues in planar imaging decreased to − 21%. Moreover, for spherical tumors with 3.6 or 2.9 cm diameters, the activity could be undervalued by − 6% to − 47%, depending on the tumor site^60^. In regard to optimizing dosimetry techniques, Loudos et al.^61^ recommended employing medium-specific dose absorption factors that are deduced from the water DPK and CT images. Voxel-based dose correction was implemented using medium-specific dose absorption factors. Dieudonn´e et al.^62^ carried out voxel-level density correction for heterogeneous tissues in abdominal regions employing a factor that consisted of the ratio of tissue density to voxel density. However, this approach was not validated for highly heterogeneous regions. Another approach, recently adopted by Khazaee Moghadam et al.^63^ applied tissue-specific DPKs, for different media (including bone, lung, adipose, and breast) applied to the mathematic Zubal phantom and compared with the water DPK and direct MC methods for several isotopes. The results presented enhancement over the conventional water DPK method. Lee et al. performed internal dose estimation by determining multiple voxel-wise S-values for different tissues with different densities. Subsequently, each density-specific dose map was thereafter multiplied by the related binary mask of the specified density tissues derived from the CT images which led to the estimation of the dose distribution maps by superimposing the multiple density-specific dose maps. This approach enhances the precision of the dosimetry calculation procedure in contrast to the single voxel S-value method but it principally assumes that the deposition of energy in each voxel results from self-absorption^19^. As shown in Fig. 6, our proposed method relatively agreed with the MC. Some false estimates were observed at lungs and spleen. The errors in spleen might be due to its small dimensions preventing the accurate segmentation. Lung regions possess large motion artifacts, and since our intention in lung dosimetry is mainly tumors that are placed inside organs, wrong calculation at the lung boundaries may not be an important problem. We confirmed that the multiple DPK approach could facilitate personalized dosimetry at the whole-body level with reasonable accuracy.

AI techniques have been developed to attend to multifarious problems by rendering the underlying physics of the issue into the realm of machine vision^64^. In this study, we developed the idea of the DPK-based dosimetry method of planar imaging by applying four different AI approaches including SVR, MLP, decision tree, and linear regression to estimate organ-specific doses. In addition to the above-mentioned four training models, we also tested CNN and U-Net algorithms; nevertheless, the results were irrelevant and thus we decided to discard them. Based on our results, the MLP network could be better fitted to the data of dose prediction. Figure 6 illustrates the variation between the MLP-based dose map and the ground truth data. For most of the body tissues, we observed below 15% differences between the MLP-based dose estimation and ground truth data. According to the results, the MLP-based approach represented the largest error for the spleen and the Lung-upper and lower parts.

In a recently published study, Scarinci et al.^65^ used machine learning to provide the DPK values for beta emitters. The results reported differences lower than 7% for the absorbed dose in patient-specific dosimetry in comparison with full MC calculations^65^. In another study, Götz et al.^31^ proposed machine learning approaches like Green’s function-based empirical mode decomposition and deep learning along with the soft tissue kernel derived from MC simulations for dose estimations. They applied a hybrid approach utilizing deep U-Net architecture along with an empirical mode decomposition to efficiently predict radiation dosage in internal radiation therapy. The algorithm received CT tissue density maps and dose maps determined from SPECT distributions of ^177^Lu and predicted each individual’s absorbed dose distributions. The system was trained using each individual’s full MC simulation results as a reference. Discrepancies between the estimated dose and the MC results confirmed a superior performance of the proposed hybrid DNN-empirical mode decomposition method in comparison with its corresponding MIRD DPK dose calculation approach^31^.

The majority of previous studies that have been done so far applied deep learning algorithms on SPECT/CT or PET/CT images and therefore one important advantage of the present study is associated with the absorbed dose estimation in 2D planar images. In fact, despite the considerable accuracy and advantages of SPECT/CT and voxelized dosimetry, we used 2D planar imaging and pixel-based dosimetry to provide the opportunity to perform pre-therapeutic imaging through the administration of low activity of ^131^I before starting the radioiodine therapy and identifying the distributed dose to different organs which could be helpful for physicians to optimize the prescribed doses. This idea should be performed only 4–5 h before performing the radioiodine therapy to prevent stunning phenomena therefore performing a whole-body SPECT/CT is not possible in the limited time. Thus, we propose to compensate for the lack of SPECT/CT with 2D planar imaging as a pre-therapeutic technique to estimate the amount of correct administered dose required to more accurately destroy cancerous cells while minimizing the adverse effect on normal tissues.

The trained MLP-based predicted dose values might be a powerful means as a patient-specific dosimetry approach which could predict absorbed dose values to different organs just in a few minutes using planar images. It should be noted that the proposed method has the potential to be implemented on planar images either ^131^I-NaI or ^131^I-MIBG. Another uncertainty of the training network could be due to the simplification involved in physical models. Nevertheless, there are still some difficulties for implementation in clinical routine which might be due to the poor quality of the imaging technique. For example, accurate segmentation of small organs such as the spleen could cause considerable errors. This can be attributed to several factors, including their relatively small size, which complicates accurate segmentation, and limited spatial resolution of planar imaging. To improve dose estimation accuracy for smaller organs like the spleen and salivary glands, which are prone to errors due to limited spatial resolution, small size, partial volume effects, and superposition, we propose multiple strategies. These include employing advanced deep learning-based segmentation (e.g., attention-guided networks, Attention U-Net, or Transformers) and super-resolution techniques (e.g., SRCNN, GANs) to enhance delineation and resolution. Multi-task learning frameworks and physics-informed neural networks can improve segmentation and dosimetry by leveraging shared features and physical principles. Additionally, adaptive multi-scale dose point kernels, synthetic data augmentation, multi-view imaging (e.g., adding lateral views), and resolution recovery techniques in planar imaging can further enhance accuracy by improving organ localization, attenuation correction, and robustness for small, low-contrast structures.

Besides, the proposed optimized network was trained with the planar images of patients after administration of therapeutic radioiodine doses. However, to be applied for the pretherapeutic dose estimation purpose, it is important to use a small amount of radioiodine activity (less than 1 mci), and also the radioiodine administration should be performed only 4–5 h before the therapeutic radioiodine administration to avoid stunning the effect. Since it is not possible to carry out whole-body SPECT/CT imaging and MC dosimetry in this short period, application of AI-based dosimetry of planar imaging can be of remarkable importance. However, it should be noted that the network was not trained for such small radioiodine activities and in fact, such data did not exist which might be another important limitation of the present idea. To overcome this limitation, one suggestion might be to conduct a prospective study involving a limited number of patients who will receive tracer-level ^131^I administrations shortly (within 4–5 h) before therapy. This would provide the possibility to collect planar imaging data under low-activity conditions, better representing the clinical scenario for pre-therapeutic dosimetry. Given the limited size of such datasets, utilizing transfer learning and model fine-tuning techniques might be helpful. The present model, trained on high-activity therapeutic data, could serve as a robust foundation. By fine-tuning it using the new low-activity dataset, the model can adapt to differences in noise levels, image contrast, and signal-to-background ratios typical of tracer-dose imaging—without the need for training from scratch. This approach not only addresses the data scarcity challenge but also improves the model’s generalizability and clinical utility. Moreover, the segmentation of lungs, because of motion artifacts, might be another source of dose estimation error. Furthermore, the dataset was achieved from one institution. Therefore, to apply this method for pre-therapeutic dose estimation, it is important to test the hypothesis with a large population and different institutions and scanners. In this regard, additional validation studies might be required with significantly larger population data and diverse radiotracers.

## Conclusion

This study utilized a deep learning algorithm on a modified ^131^I planar WB imaging data as well as using additional single oblique view to estimate different organ doses during radionuclide therapy. The findings presented at least 85% accuracy in organ dose prediction using MLP, compared to the MC SPECT/CT dose mapping. Despite the established accuracy of MC voxelized dosimetry, the time-intensive calculations prevent it from being utilized in clinical routine. Therefore, modified 2D planar imaging and pixel-based dosimetry enable pre-therapeutic imaging through the administration of a low amount of radioactivity of ^131^I facilitating the organ-specific dose distribution for optimizing prescribed activities. Nevertheless, there are still challenges in the clinical integration of this method, potentially attributed to image quality and segmentation issues. Validation with a larger study population is essential for its application in pre-therapeutic dose estimation.

## Data Availability

All datasets used and /or analyzed in this study are not publicly available, but are available from the corresponding author on reasonable request.
